# Robotic-Assisted 3D Bio-printing for Repairing Bone and Cartilage Defects through a Minimally Invasive Approach

**DOI:** 10.1038/s41598-019-38972-2

**Published:** 2019-03-06

**Authors:** Julius Lipskas, Kamal Deep, Wei Yao

**Affiliations:** 1 The Department of Biomedical Engineering, University of Strathclyde, Glasgow, UK; 2 Golden Jubilee National Hospital, Glasgow, UK

## Abstract

There is an unmet need for new techniques and methods of healing critical size tissue defects, by further reduction of invasiveness in implant, cell and tissue-based surgery. This paper presents the development of a new regenerative medicine that combines 3D bio-printing and robotic-assisted minimally invasive surgery techniques to meet this need. We investigated the feasibility of Remote Centre of Motion (RCM) and viscous material extrusion 3D printing. A hypothetical, intra-articular, regenerative medicine-based treatment technique for focal cartilage defects of the knee was used as a potential example of the application of 3D printing *in vivo*. The results of this study suggest, that RCM mechanism is feasible with viscous material extrusion 3D printing processes, without a major trade-off in imprint quality. The achieved printing accuracy at an average dimensional error of 0.06 ± 0.14 mm in this new modality of 3D printing is comparable to those described in literature for other types of bio-printing. Robotic assisted 3D bio-printing demonstrated here is a viable option for focal cartilage defect restoration.

## Introduction

Future developments in medical science, regenerative medicine and materials science may allow the repair of human body parts using 3D bioprinting techniques and serve as a basis for new therapies for tissue and organ regeneration.

Although a substantial number of studies focused on 3D printable bio-scaffold development^1–6^ can be found in the literature, surprisingly, very few of them have focused on potential *in vivo*/*in situ* delivery options, which may prevent the time-consuming stage of scaffold preparation and reduce the risks of scaffold contamination.

On the other hand, studies that consider such a possibility^7–10^ employ 3D printing manipulators, which have three translational degrees of freedom, i.e., Gantry-type manipulators. Although this type of robotic device could potentially be used *in vivo*, it is clearly more feasible with an open surgery than a more advanced minimally invasive surgery (MIS), which has many additional benefits, including better safety, decreased scarring, faster recovery and shorter hospital stay − the results of less traumatic surgical intervention^11^.

We hypothesise that in the future, knee cartilage defects could be treated by *in vivo* 3D printing from biological/biocompatible materials to produce a suitable cell attachment and proliferation environment in the damaged site and employ the natural human body recovery potential. Several techniques using such an approach have been reported in the literature, including autologous chondrocyte transplantation^12^ and autologous collagen-induced cartilage repair techniques^13^. Although these methods at the current stage of development may not be suitable for the repair of end-stage osteochondral defects, where implants are required not only because of articular surface defects but also for restoring normal knee joint geometry and limb alignment, they are routinely used for healing limited volume damage to bone and cartilage in the joint, such as localized focal osteochondral or chondral lesions. In addition, the development of regenerative medicine and tissue engineering-oriented arthroplasty techniques may contribute to the knowledge of the entire field. This in turn could lead to better articular disease treatment techniques and solve the clinical problem of healing critical size articular osteochondral defects^14^.

Previously mentioned techniques were executed manually; however, there are no obvious obstacles to performing these techniques using robotic manipulators and MIS approaches, for example, using the robotic bone milling process for site preparation and the extrusion 3D printing process for damage repair material applications. In addition, this will lead to more accurate site preparation and imprint placement, which presumably could be achieved using robotic manipulators^15,16^. This will have a positive influence on treatment effectiveness as well as minimise the risk of scaffold contamination. In the proposed surgery technique, knee portals used in arthroscopic surgery could be employed as the entry points for robotic manipulator end effectors to reach required parts of the knee. The remote centre of motion (RCM) operating specifics could lead to avoiding large cuts and associated negative effects.

In this study, we aim to evaluate a novel idea for regenerative medicine, based on robotic MIS and 3D-bioprinting. To investigate the feasibility of the RCM mechanism, which is traditionally used in robotic-assisted MIS, operation and viscous material extrusion 3D printing processes we attempted to restore an osteochondral defect and evaluated effectiveness of this novel approach.

A newly developed RCM mechanism-based robotic system that was used for touch probe scanning, creating an osteochondral defect by milling, and later for restoring the articular surface with a hydrogel 3D printing process. The material of choice for these experiments was a photocurable alginate-poly(ethylene glycol) diacrylate hydrogel, which according to its developers is 3D printable, has excellent biocompatibility and can be tougher than natural human cartilage when mixed with nanoclay^17^.

## Methods

### RCM manipulator construction

**Figure 1:**
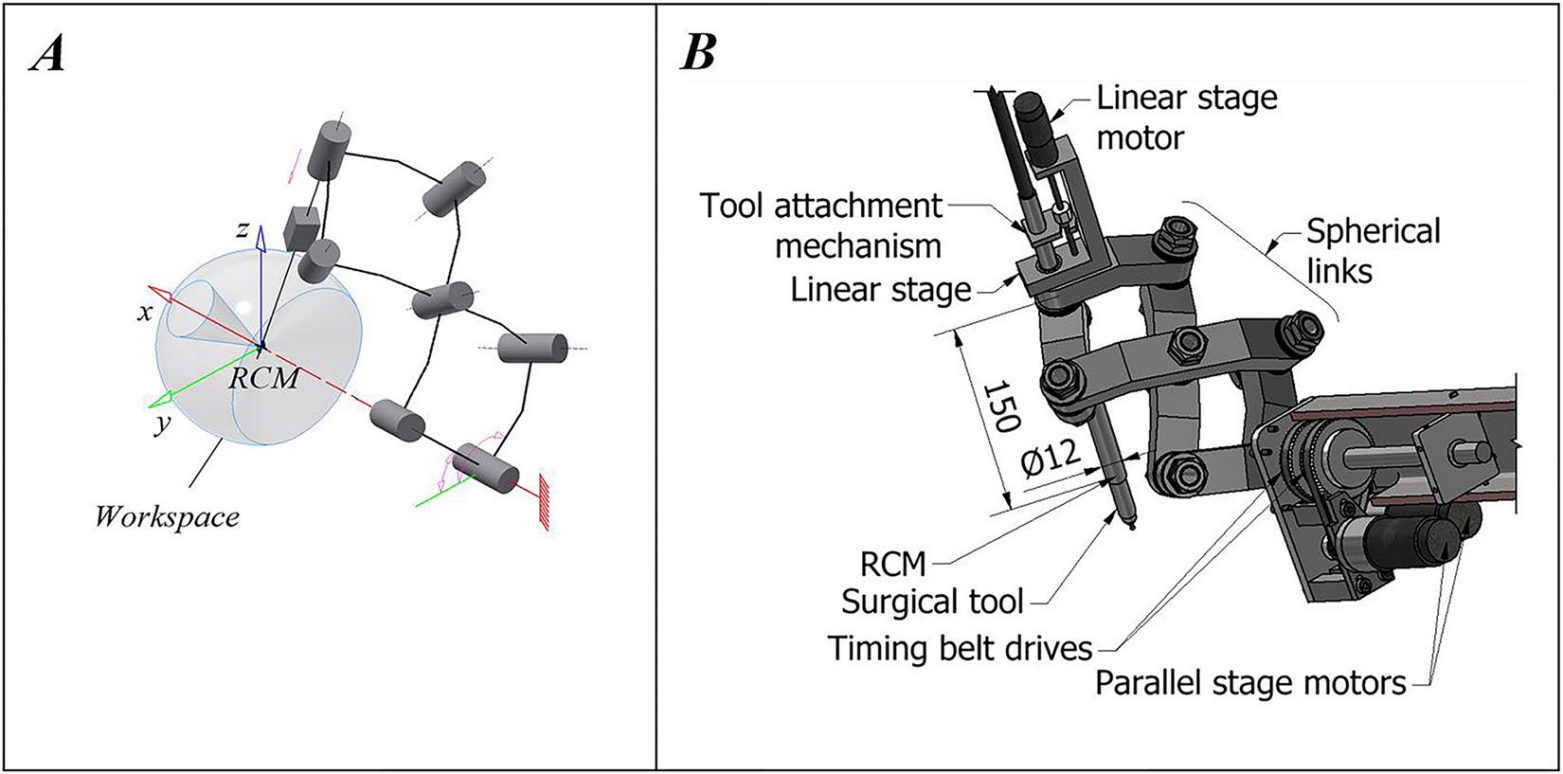
Robotic device construction. **(A)** Kinematic schematics of the RCM mechanism. **(B)** CAD model of the RCM mechanism.

**Figure 2:**
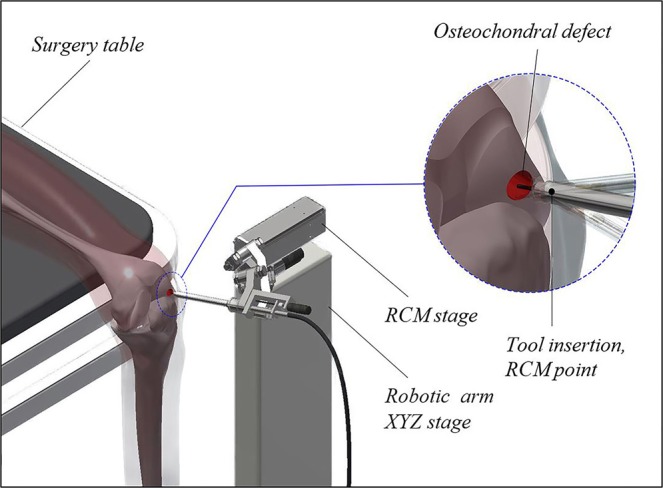
Proposed surgery setup.

The RCM manipulator consists of a parallel manipulator stage, formed by six spherical links, assembled in a pentagram-like structure to increase the range of rotation about the Y axis; an independent linear stage; and hardware and firmware for its control. Kinematic schematics and the CAD model of the manipulator are shown in Fig. 1A,B, respectively. The manipulator is capable of rotating 360° around the X axis and ± 40° around the Y axis, has a 70 mm linear stage extension and has a no-load positioning repeatability of 0.15 mm, the bulk of which might be associated with an acceptable absolute position error limit of 0.1 mm between the target and reached coordinate in the custom control firmware. A manipulator of this type could be attached to an XYZ stage for the initial positioning of the RCM at the knee entry portal. An illustration of the proposed surgery setup is shown in Fig. 2.

**Figure 3:**
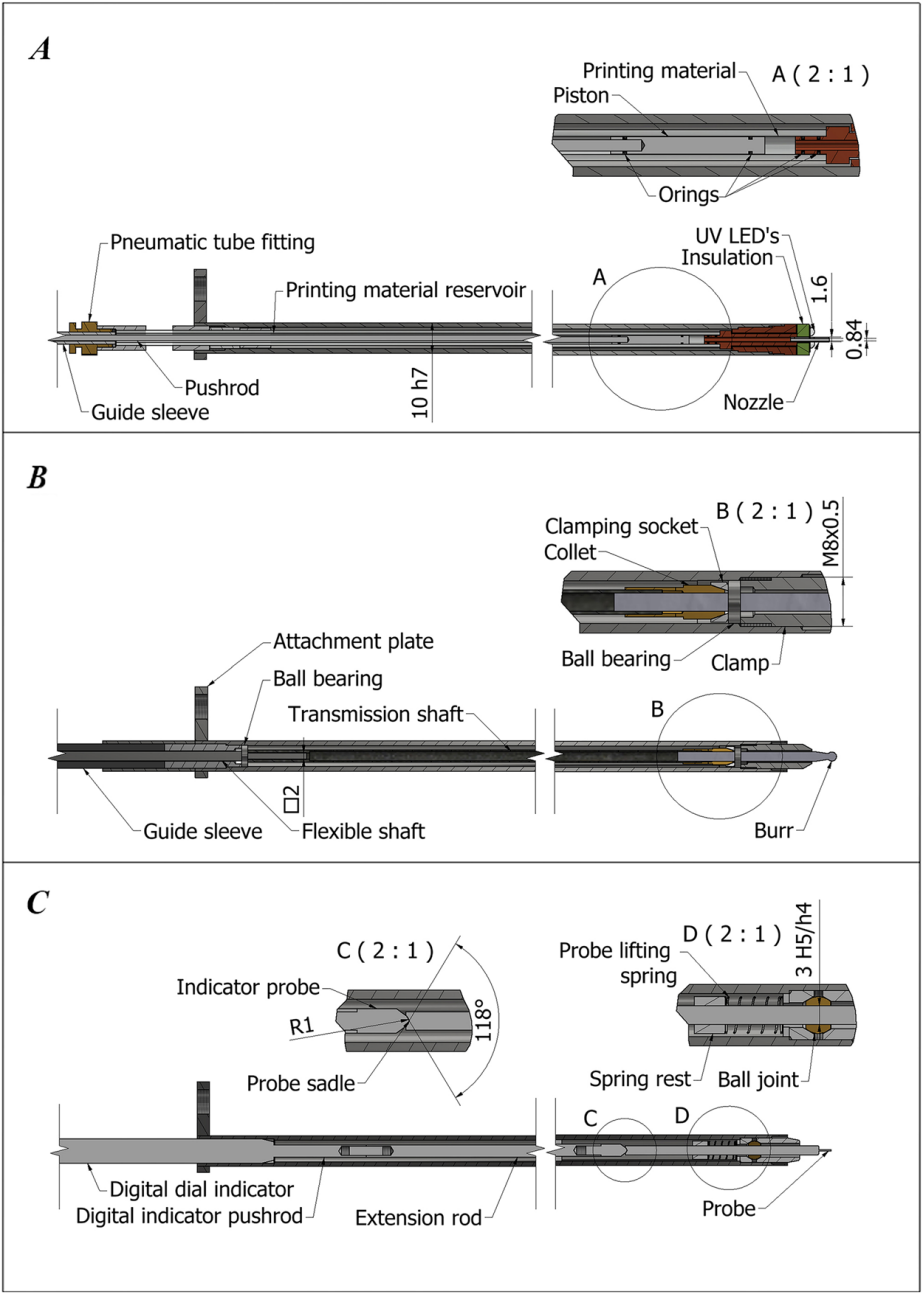
Construction of robotic device end effectors **(A)** 3D printing tool **(B)** Bone milling tool **(C)** Touch scanning probe.

The manipulator can currently handle three rapidly interchangeable end effectors for bone milling, 3D printing and a digital industrial indicator-based touch probe. An illustration of the end effectors can be seen in Fig. 3.

The manipulator is controlled using an Arduino Mega (Arduino, Italy) programable microcontroller and a custom control firmware. A universal asynchronous receiver-transmitter protocol is used for communication between the controller and a personal computer (a standard Arduino feature). The control code utilises the staircase interpolation algorithm^18^ for path generation.

In addition, the device can be controlled using a custom-made control panel that consists of a manual control enable/disable switch, six push buttons to indicate the direction of movement and three potentiometers to adjust the speed of rotation of each of the motors. It enables one to move each of the motors manually, while the controller sends calculated end effector position coordinates through the communication interface with a personal computer.

### Preparation of the bone/cartilage substrate

**Figure 4:**
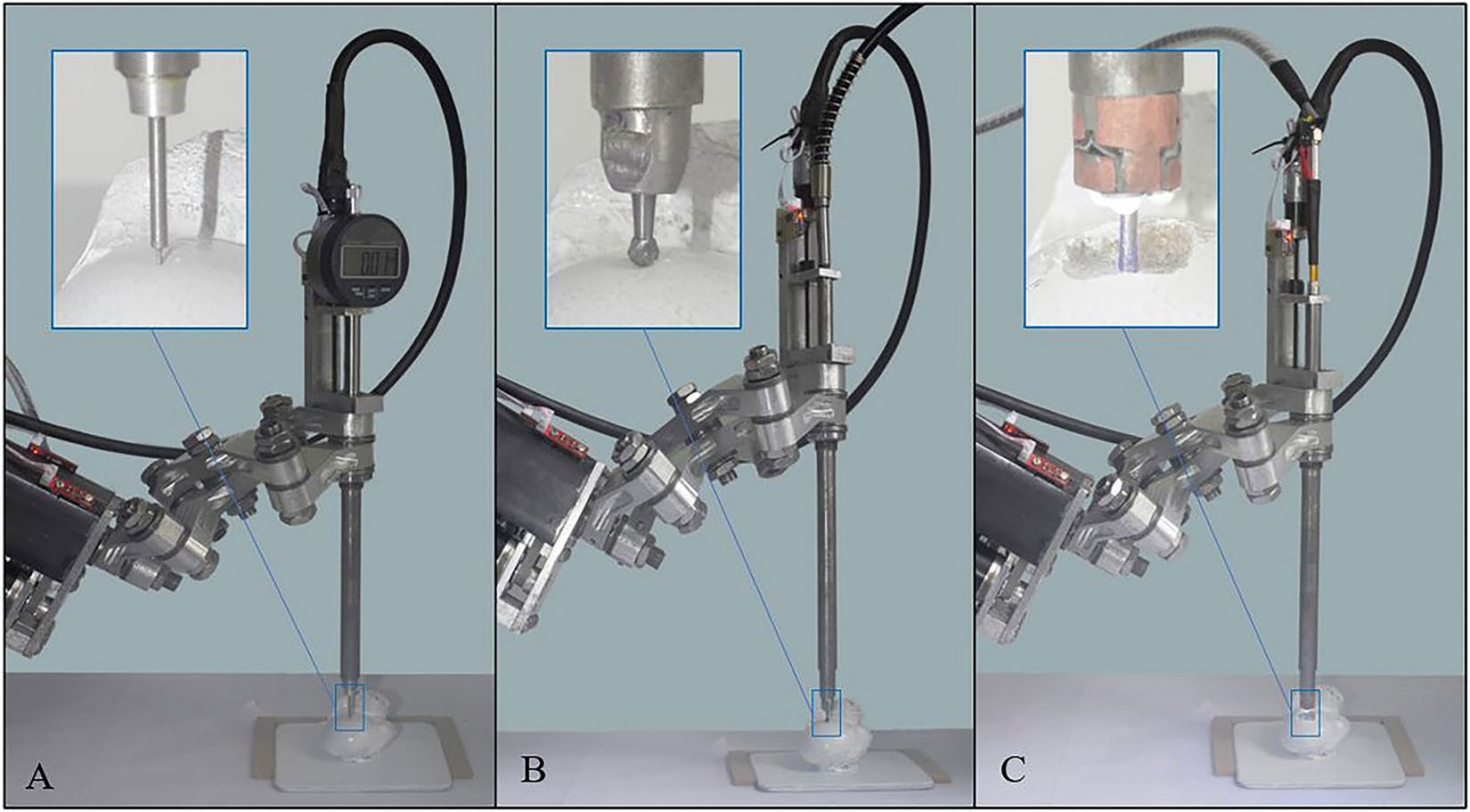
Experimental setups for **(A)** surface registration, **(B)** bone milling and **(C)** 3D printing.

A freshly harvested ovine humerus was obtained from a local butchery. The remaining soft tissues were removed without altering the geometric parameters of the humerus head, and the bone was placed in boiling water for 10 minutes to disinfect and preserve the bone. The humerus was then cut approximately 50 mm from the proximal end and attached to a piece of acrylic plastic with two wood screws. Finally, the assembly was attached to the workbench in reach of the end effector of the manipulator using industrial double-sided sticky tape together with plastic reregistering planks for easier placement of the substrate in this same position after subsequent 3D scanning steps. Specimen positioning can be seen in Fig. 4.

### 3D scanning

The bone substrate was scanned using a Konica Minolta Vi-9i 3D scanner, which has a maximal accuracy of ±0.05 mm and outputs coordinates of 307200 points per single scan^19^. Prior to scanning, the sample was coated with white spray paint (ITP Imports, United Kingdom) to increase the surface light reflection and, hence, to achieve better scanning performance. The highest scanning resolution was chosen for all of the scans. Twelve scans per sample were performed, which were later imported to Geomagic Wrap software (3D Systems, USA) and constructed into a single WRP file for further processing and printing accuracy evaluation.

### Substrate registration and defect volume modelling

The surface of interest of the substrate was registered to the manipulator coordinate system, and its geometrical properties were measured using a manual control interface of the RCM manipulator and touch probe attachment. The touch probe was mounted on the manipulator, and its position was registered using an autocalibration function in the manipulator’s firmware. Upon the confirmed contact of the end effector in each measurement, resultant cartesian coordinates were manually entered in a MS Excel file and were later used to recreate surface features in Autodesk Inventor (Autodesk, USA) software by creating a cluster of 3D points, joining them with the required number of sketches and forming a solid 3D body using the software’s “Sweep” feature. The touch probe scanning setup is shown in Fig. 4(A). The created solid was later used for G-code generation for bone milling and 3D printing.

### Bone milling

To create an osteochondral defect on the bone-cartilage substrate for the following 3D printing procedure, the bone milling attachment was mounted on the manipulator, and its position was registered using the autocalibration function in the manipulator’s firmware. The G-code for this procedure was written manually based on the previously obtained solid volume of the intended defect. The bone milling setup is shown in Fig. 4(B).

A solid tungsten carbide 3 mm fluted ball nose and 3 mm shank cutter was used as a bone cutting tool (Burr shape D30303-2, Aria Developments Ltd., UK). The G-code was sent to the manipulator controller using Repetier Host (Hot-World GmbH & Co. KG, Germany) software. A cutting tool feed of 2.5 mm/s, cutting depth of 1 mm, rotational speed of 15000 rpm, and cutting width of 1 mm were selected for this experiment. After the milling procedure, the defect was cleaned and dried using di-methyl ether-based air duster (ITW Contamination Control BV, USA). The milled defect dimensions were verified using machinist’s callipers by taking a series of five approximately equally spaced measurements in each direction.

### Preparation of the hydrogel

Alginate-poly(ethylene glycol) diacrylate hydrogel was prepared using a technique described by Li *et al*., in which “… two solutions consisting of 6% w/v sodium alginate powder and 80 mM CaCl_2_ were mixed with a volume ratio of 1:1 to result in a partially crosslinked hydrogel. Then, 10% w/v PEGDA and 0.05% w/v I-2959 were added to provide a cytocompatible photoinitiating conditions.”^8^ All required materials were obtained from Sigma Aldrich, USA. After mixing, the material was loaded into the reservoir of the printing nozzle attachment and degasified.

### 3D printing

The G-code for the 3D printing operation was obtained using Repetier Host software and an STL file of a previously obtained osteochondral defect model. A first layer height of 0.25 mm, layer height of 0.1 mm, infill density of 100%, printing nozzle feed of 3 mm/s and printing nozzle size of 0.84 mm were selected for this experiment. In addition, the G-code was manually modified to exclude unnecessary/not currently supported commands (all commands with F operation codes) and later sent to the manipulator controller using Repetier Host software.

The 365 nm ultraviolet (UV) light required for material curing was delivered by three light-emitting diodes (NCSU276AT-0365, Nichia, Japan). The UV light intensity was verified using a hand-held optical power meter (PM160T, Thorlabs, USA) and found to be 39 mW/cm^2^ at 365 nm, in plane, perpendicular to the longitudinal axis of the attachment, at the tip of the nozzle.

The 3D printing attachment was mounted on the manipulator, and its position was registered using the autocalibration function in the manipulator’s firmware. The 3D printing setup is shown in Fig. 4(C).

UV light was used during the entire printing process, as well as 10 minutes after, for fully curing the most superficial layers of the print.

### Printing accuracy evaluation

Files of the initial, intact bone specimen and the specimen with restored defect were analysed using Geomagic Wrap software. Previously obtained WRP files of complete scans were imported into the software and registered using the global registration function in the software interface. The area of interest (20 × 20 mm cut around the bone defect) was isolated and later compared in terms of mesh-to-mesh distance. The results of the comparison were obtained as colour scale maps. In addition, the exact area of the defect was also isolated and compared to obtain a list of main statistical parameters.

## Results

### Surface registration

**Figure 5:**
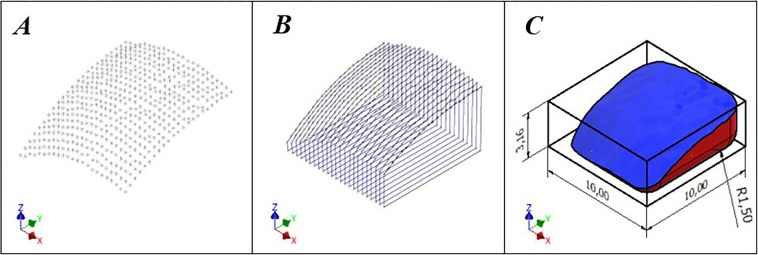
Defect volume modelling steps using Autodesk Inventor software. **(A)** Touch probe contact positions as a cluster of 3D points. **(B)** Series of created slice profiles. **(C)** Solid of intended defect volume.

In total, coordinates of 441 points, approximately 0.5 mm apart, were obtained at the specimen surface area of interest (10 × 10 mm area on the proximal end of the humerus head) and later successfully used to recreate the defect volume. In addition, the volume was trimmed to the intended dimensions, and the corners were rounded by placing a 1.5 mm fillet (radius of the burr) on the bottom and vertical edges. The main steps of the volume modelling process are shown in Fig. 5.

### Osteochondral tissue milling

**Figure 6:**
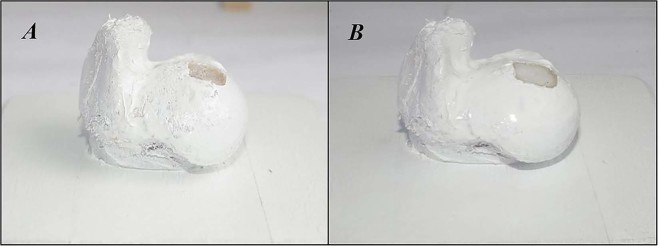
Bone specimen before and after the 3D printing procedure. **(A)** Specimen with milled defect. **(B)** Specimen with hydrogel infill.

Visual inspection of the created osteochondral defect milling revealed a clean surface of the cut, without visible imperfections, that had the intended shape and sharp, undamaged corners on the surrounding tissue (Fig. 6(A)). The largest dimensional error was found to be + 0.2 mm in the central part of the defect width (Y axis direction in Fig. 5).

### 3D printing

Visual inspection of the 3D-printed osteochondral defect infills revealed a smooth superficial surface, with seemingly correct dimensions and geometrical placements (Fig. 6(B)).

The volume of the defect of 210 mm^3^ (Autodesk Inventor output) was filled in 645 s, giving an average material application rate of 0.33 mm^3^/s.

**Figure 7:**
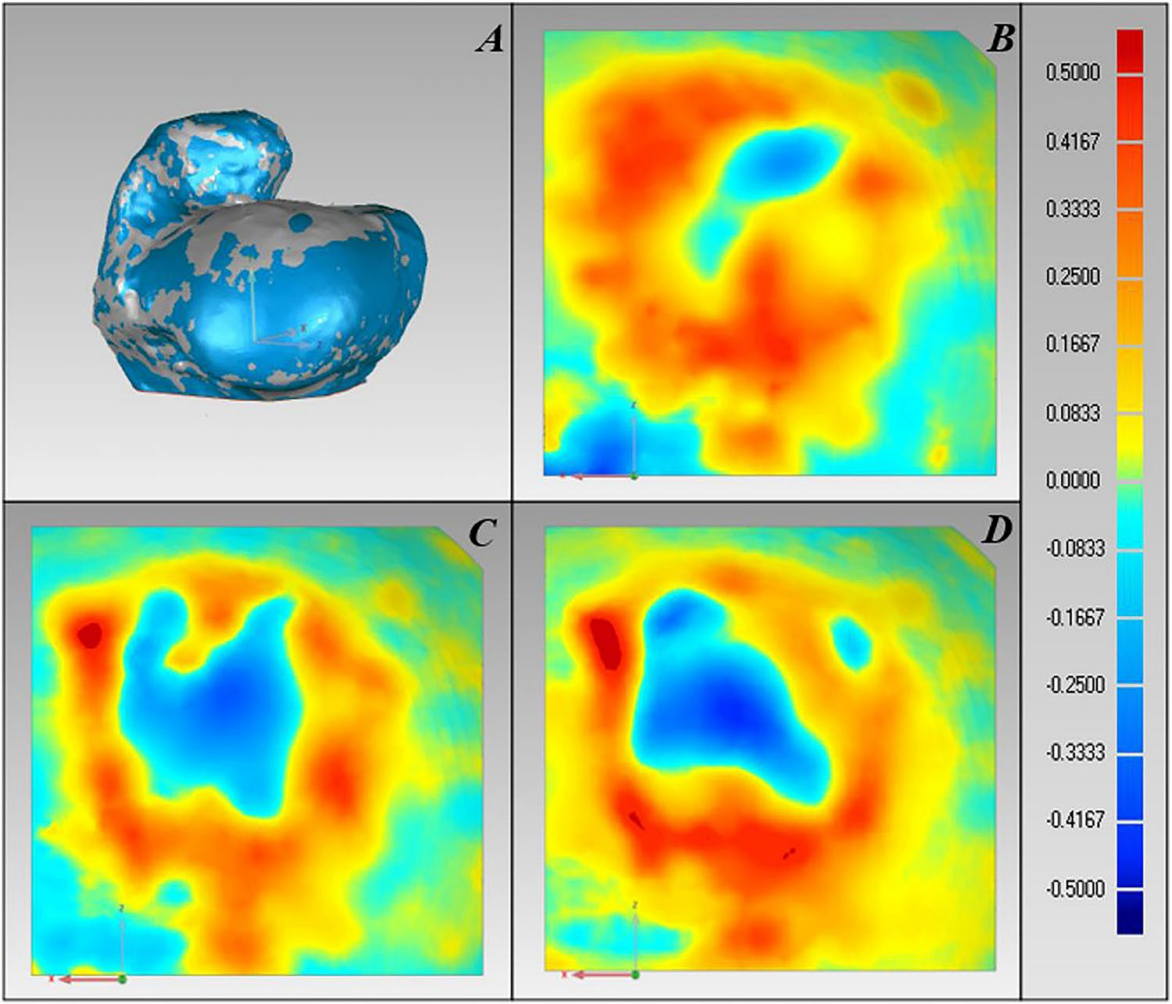
Comparison of scanned 3D samples in Geomagic Wrap software. Imprint dimensional error magnitude colour scale is applicable to all colour maps; one unit represents 1 mm. **(A)** Registered clouds of two samples. **(B)** Sample-to-sample distance colour map of restored defect area, imprint 1. **(C)** Sample-to-sample distance colour map of restored defect area, imprint 2. **(D)** Sample-to-sample distance colour map of restored defect area, imprint 3.

The results of the 3D scans of the intact substrate and the substrate with restored defect are summarised in Fig. 7. Dimensional error magnitude maps, together with the main statistical parameters (Table 1), show consistent errors between all imprints, with a small negative error near the centre of the print and a positive error of larger magnitude closer to the imprint borders, as well as some printing material deposition outside the printing space.

**Table 1:** Printing accuracy evaluation, main statistical parameters.

Print No.	Error interval, mm	Average error, mm	Standard deviation, mm
1	−0.46 to 0.50	0.10	0.14
2	−0.37 to 0.59	0.06	0.14
3	−0.45 to 0.59	0.03	0.16

The only technical issue noticed during the printing process was a slight sticking of extruded material to the tip of the printing nozzle and related viscous infill volume drag/infill deformation.

## Discussion

In terms of imprint accuracy, the results of this experiment, with an average defect imprint geometrical error of 0.06 ± 0.14 mm are comparable to those reported in the literature of 0.1 ± 0.1 to 0.46 ± 0.32^7,8^. The implant placement errors reported here are within the accuracy requirements for the Osteochondral Autologous Transfer System (OSTS) surgery procedure for osteochondral defect repair of ± 0.6 mm, although cartilage grafts implanted beneath the articular surface are generally unacceptable^7^. The negative dimensional errors near the centre of the print are likely associated with a scraping action of the nozzle corners, which occurs due to rotation of the tool around the RCM, while excessive material application towards the border of the print clearly correlates with the nozzle inclination angle. On the other hand, the consistent error magnitude and variation among all prints may suggest that these deficiencies occurred due to systematic error, and therefore, corrective action can be expected to be straightforward in the process optimisation stage.

However, the creation of dedicated or optimised current nozzle path generation algorithms for this new modality of 3D printing might be necessary to compensate for nozzle inclination effects and to decrease dimensional errors.

Noticed sticking of extruded material to the tip of the printing nozzle and related viscous imprint volume drag/infill deformation, may suggest that the 3D printing attachment for this type of material would benefit from a hydrophobic (i.e., polytetrafluoroethylene) coating, which is likely to reduce non-solid material attachment and may increase 3D printing process accuracy and prevent material deposition outside the printing area.

Experiment results suggest, that RCM mechanism kinematic structure-related aspects of operation are feasible with viscous material extrusion 3D printing processes without a major trade-off in imprint quality.

The current *in vitro* printing of scaffolds not only requires additional steps and equipment but also predisposes the risk of contamination. The size of the incision must be larger than our proposed technique due to surgical difficulty, and the scaffold may also need another glue medium for sticking to the implantation site. In our technique, this is avoidable because printing occurs at the site of implantation, there is no transport involved, and the printed scaffold can cure at the site of implantation itself. The robot can create an inherently stable shape configuration at the site of implantation, and thus, on-site printing becomes inherently stable. Minimally invasive techniques have also proven to be less painful postoperatively, leading to early discharge and rehabilitation, endowing the patient with increased early function and helping the hospital save money due to reduced bed occupation. Our proposed technique should be able to take the advantages of both. Moreover, similar tissue defect restoration could potentially take place in other organs.

A major issue, which must be solved prior to creating *in vivo* 3D printing-based therapies, is the lack of reliable and harmless in-surgery navigation techniques. Robotic systems for orthopaedic surgery that are currently on the market utilise pre-surgery computed tomography scans and in-surgery optical tracking of bone-attached markers (MAKO RIO Robotic Arm, MAKO Surgical Corp., Fort Lauderdale, FL, USA^20^) or mechanical tracking, which require invasive registration of articular surface landmark points (Acrobat Sculptor, Stanmore Implants, Elstree, UK)^21^. However, the disadvantages of such approach include additional injury due to optical tracker attachment, more invasive procedure, ionising radiation dose during preoperative scanning and reduced accuracy (errors can be up to 1 mm when practically using tracker-based navigation systems). This could diminish the potential benefits of the presented more accurate, less damaging approach, that has less chance of potential risks associated with *in vitro* scaffold printing techniques.

Touch probe-based imaging techniques are being used in the medical field, mostly in the dental industry^22^, and are known to be one order of magnitude more accurate than the OSTS requirements^23^, which in turn could make errors of previous surgery stages negligible. The scanning process, which we successfully used for geometric data acquisition and subsequent modelling of osteochondral defect modelling, has the potential for use as a basis for orthopaedic surgical navigation/data acquisition/registration of the anatomy. Such method is presumably relatively harmless and avoids the use of ionising radiation. However, the navigation system is not in the scope of this work, and we used this approach mainly to exclude the influence of experimental variables associated with potential navigation system errors, as well as those that may occur due to the not yet fully characterised accuracy of our robotic manipulator, and to rely only on the already known repeatability of the device.

Alginate hydrogels are some of the most widely used biomaterials in 3D bioprinting. The materials used in this work, in addition to the partially crosslinked alginate hydrogel, include a combination of PEGDA and 2-hydroxy-4′-(2-hydroxyethoxy)-2-methylpropiophenone, which is known to be mildly cytotoxic^24^. Moreover, 2-hydroxy-4′-(2-hydroxyethoxy)-2-methylpropiophenone, which is responsible for UV-induced crosslinking, is not a fully tested substance^25^ yet, which may complicate application of this particular hydrogel material *in vivo* in humans in very near feature. On the other hand, requirements for the printing material in this printing technique are similar to those of other methods, which in turn allows choosing from a wide range of biocompatible inks.

Bone substrate preparation using the bone milling process was executed successfully, and at the largest dimensional error of +0.2 mm, the preparation quality is unlikely to significantly influence the results of this work.

With current hardware, the system could potentially access only the most superficial part of the knee; therefore, additional hardware, such as flexible tipped tools^26^, might be necessary to access deeper parts. However, even current accessibility range is similar to that of current treatments, since they utilise similar joint space entry ports and straight shank tools.

Typical *in situ* 3D printing material deposition rates cannot be found in the literature at this time; however, osteochondral lesions with a typical area of 3.2 cm^2 ^^27^ at a current material application rate of 0.33 mm3/s should finish surgery in a reasonably quick time. In addition, this parameter is highly adjustable.

## Conclusion

In this work, we investigated the feasibility of RCM-based robotic system and viscous material extrusion 3D printing using a hypothetical, intra-articular, regenerative medicine-based treatment technique for focal cartilage defects of the knee, as a potential example of the application of our technique for 3D printing *in vivo*.

This approach could allow utilisation of the benefits of MIS, minimise the risk of scaffold contamination in regenerative medicine, and help eliminate the *in vitro* scaffold preparation step. To the best of our knowledge, this is the first documented attempt to use the RCM mechanism for 3D printing.

Robotic-assisted 3D bioprinting for regenerative medicine using bioactive/biocompatible/cellular materials, demonstrated here, is a viable option for focal cartilage defect restoration. This in turn could lead to further reduction of invasiveness in the arthroplasty and may allow the restoration of other organs *in situ* in the future.

## Data Availability

All data underpinning this publication are openly available from the University of Strathclyde KnowledgeBase at 10.15129/261432c4-ad25-47e4-a5fa-a3420e35a76c.
